# Experiments on torrefied wood pellet: study by gasification and characterization for waste biomass to energy applications

**DOI:** 10.1098/rsos.150578

**Published:** 2016-05-25

**Authors:** Andrew N. Rollinson, Orla Williams

**Affiliations:** Department of Engineering, University of Nottingham, Energy Technologies Building, Innovation Park, Triumph Road, Nottingham NG7 2TU, UK

**Keywords:** gasification, biomass, torrefaction, waste to energy, kinetics, grindability

## Abstract

Samples of torrefied wood pellet produced by low-temperature microwave pyrolysis were tested through a series of experiments relevant to present and near future waste to energy conversion technologies. Operational performance was assessed using a modern small-scale downdraft gasifier. Owing to the pellet's shape and surface hardness, excellent flow characteristics were observed. The torrefied pellet had a high energy density, and although a beneficial property, this highlighted the present inflexibility of downdraft gasifiers in respect of feedstock tolerance due to the inability to contain very high temperatures inside the reactor during operation. Analyses indicated that the torrefaction process had not significantly altered inherent kinetic properties to a great extent; however, both activation energy and pre-exponential factor were slightly higher than virgin biomass from which the pellet was derived. Thermogravimetric analysis-derived reaction kinetics (CO_2_ gasification), bomb calorimetry, proximate and ultimate analyses, and the Bond Work Index grindability test provided a more comprehensive characterization of the torrefied pellet's suitability as a fuel for gasification and also other combustion applications. It exhibited significant improvements in grindability energy demand and particle size control compared to other non-treated and thermally treated biomass pellets, along with a high calorific value, and excellent resistance to water.

## 1. Introduction

Biomass is a potentially sustainable energy source only in certain circumstances: where new plant growth equals the rate at which it is removed (hence maintaining the atmospheric CO_2_ balance) and where no fossil fuels have been used in fertilization, processing and transportation. For this reason, in a hierarchy of options related to the sustainable applications of biomass, locally sourced waste (such as low or negative value forestry residue) is considered to be at the top and dedicated energy crops are at the bottom [1]. Dedicated bio-energy crops are primarily supplied to large centralized power stations, where due to market demands, the biomass is dried in fan-assisted ovens [2,3], transported long distances (often from different continents) [4], and almost always pelletized [5], all of which undermine its sustainability credentials. Utilization of locally sourced biowaste for energy however—currently part of a directive to avoid landfill—necessitates only short transportation distances because of the close proximity to end use. As this resource is a by-product of sustainable land management, no petrochemical fertilizers need be used in its culture, and because of the relatively low volumes required, passive drying (seasoning) is also feasible [1].

Small-scale downdraft gasification is a technology that has been used for over a century to generate electricity and provide motive power from locally sourced waste biomass [6–11]. Gasifiers provide energy on demand so battery storage is unnecessary; and there is no working fluid, so the systems offer improved efficiencies compared to traditional combustion/steam turbine generators [7]. Furthermore, NO_*x*_ and particulate emissions are reportedly lower from gasifier engines, likely due to the way that the engine is adjusted to cater for this type of fuel [12,13]. The likelihood of dioxin production in a gasifier is less well defined, but recent tests found that 30–60% of the biomass chlorine content remained in the char, and that dioxin precursor formation was inhibited by the restricted O_2_ levels inherent within the process [14].

Interest in gasification has recently returned and small-scale systems are now considered to have an expanding niche market [15]. This is due to a number of factors: the desire to reduce greenhouse gas emissions, obtain future energy security and manage waste; but equally because gasification can supply energy independent of seasonal or diurnal weather variations, and provide reliable energy in parts of the world that are off grid or subject to intermittent grid outages. Along with this, the technology is modular, scale-able and repairable ‘in the field’ [16,17].

In theory, anything solid and carbonaceous can be gasified, and therefore converted to energy at point of use. Yet, the technology is not currently at this stage, as the following extract relates [18, p. 15]:

One hundred years of gasification research and commercial applications have clearly shown that the key to successful gasification is a gasifier specially designed for a particular fuel. It is of paramount importance that the physical and chemical characteristics of the fuel do not change significantly.

One reason that gasifiers are not presently generic waste conversion systems is that the feedstock needs to be robust and retain its form inside the reactor to ensure adequate heat and gas transfer [19,20]. If this feedstock falls apart due to moisture absorbance or attrition, then gas circulation inside the reactor will be inhibited and the product will be overly contaminated with tar and particulates [7,20]. Pelletization is used to homogenize the fuel supplied to other energy conversion technologies and improves feeding characteristics. But whereas large-scale combustors and small-scale biomass boilers benefit from pelleted feedstock, gasifiers cannot use this type of pressure compacted reconstituted fuel. Standard commercial wood pellets, made from sawdust for example, swell when coming into contact with water each time the gasifier is shut down [21]. This was not mentioned in one recent study where a previously empty gasifier—a precursor to the model used in this study—was run with wood pellet, then emptied afterwards—a completely impractical way of operating a closed-top gasifier as it necessitates a full reactor clean out after every shut-down [22]. Feedstock physical properties also determine internal mass transfer such as its ability to flow rather than ‘bridge’ within the hopper and auger feeder [6]. It is for these reasons that the potential of gasification has yet to be fulfilled.

Torrefaction has been suggested as a possible pre-treatment method to improve the durability of reconstituted or non-woody biowaste to make it useable for gasification applications [23,24]. For bio-wastes to be used as biofuels, they cannot contain heavy metals or halogenated organic compounds [25], and the same requirements are necessary for thermally treated materials. If successful, torrefaction could markedly increase gasifier system uptake by making use of a greater variety of biomass waste. Torrefaction involves ‘roasting’ the biomass pellet at moderate temperatures of 230 ≤ °C ≤ 300. This reduces its moisture content (hence therefore increases calorific value) and also imparts a texture that is more akin to coal [24]. Despite this, no published works appear to have reported actual tests to prove or disprove the efficacy of torrefied wood pellet in a downdraft gasifier, nor are there any available reports on torrefied pellet gasification reaction kinetics.

Studying the underlying reaction kinetics of specific thermal decomposition reactions is important as it permits elucidation of both the energy requirement and reaction rate—the chemical rather than physical considerations of reactor engineering. This has specific relevance to a gasifier system, because it helps to understand how to overcome the present inflexibility with respect to feedstock heterogeneity [6,13,26,27].

At present, torrefied biomass pellets are also attractive to other energy technologies. In this context, in addition to the better handling and storage properties, one of the main drivers for torrefaction is to improve feedstock grindability, e.g. how easily the pellet will yield to mechanical stress [28]. This is important for larger applications where in addition to the pre-processing already described (when supplied to centralized power station combustion burners), the pellets are ultimately pulverized [29], thus imposing an additional energy demand and further reducing both efficiency and overall process sustainability. For biomass to be used in a pulverized fuel burner, the standard mill classifier setting is 1 mm [30,31]. Other types of industrial energy systems also require pulverized feedstocks, such as entrained flow gasifiers [32]. While no standard grindability tests exist for biomass, the two main coal grindability tests of Hardgrove Grindability Index (HGI) [33,34] and Bond Work Index (BWI) [35] have been used for characterization. The HGI test is based on Rittinger's theory that ‘the work done in grinding is proportional to the new surface produced’ [36], and lower HGI values indicate a material that is harder to grind. BWI is based on Bond's theory that ‘the net energy required in comminution is proportional to the total length of the new cracks formed’ [29], and lower BWI values indicate less resistance to milling. Ohliger *et al.* [37] found that the improvement in grindability depended on the degree of torrefaction, and the use of HGI alone could provide misleading results. Williams *et al.* [38] used the standard HGI and BWI test to analyse a range of biomass samples and found that thermal pre-treatments do significantly improve the grindability of biomasses. Van Essendelft *et al.* [39] developed a hybrid work index to analyse the grindability of torrefied pine chips and found that it was useful in assessing torrefaction and potentially correlated with industrial scale grinding energy.

Particle size analysis is also often used as an indicator of biomass grindability. Satpathy *et al.* [40] used microwave irradiation (which involves shorter residence times and permits lower temperatures) to torrefy wheat and barley straw in a batch process. They analysed the particle-size distributions as per Bridgeman *et al*. [41] to assess grindability and saw a significant improvement in grindability through torrefaction, along with the creation of a more hydrophobic pellet. Arias *et al*. [42] also used particle size distribution analysis to assess the grindability of torrefied eucalyptus chips (less than 5 mm) and also observed an improvement in grindability. Phanphanich *et al*. [43] torrefied pine chips and used energy consumption and particle size to quantify the impact of torrefaction on grindability. Specific energy consumption required for grinding reduced dramatically with an increase in torrefaction temperatures, along with the mean particle size of the ground samples. Repellin *et al*. [44] also noted a reduction in particle size for wood chips torrefied in a pilot kiln. Adapa *et al*. [45,46] used steam explosion to torrefy barley, canola oat and wheat straw and found increased particle densities, reduced specific energy consumption, and reduced particle size and bulk density as a result of torrefaction. For straw, the electrical demand of microwaves is around 1.3 MJ kg^–1^, with the heat and conversion loss of electricity to thermal energy at around 42% [47], and torrefied biomass retains 75–95% of its original energy content [48]. From a techno-economic perspective, torrefied pellets offer cost savings over wood pellets as a coal replacement fuel. Despite slightly higher production costs, torrefied pellets have a cost price of 9.8 USD/GJ for torrefied pellets compared with 12.8 USD/GJ for wood pellets, and are one of the most cost-effective options for CO_2_ mitigation [49].

However, most of these studies focus on the torrefaction of coarse feed materials prior to densification. As Temmerman *et al*. noted [50], most biomass grinding studies focus on non-densified samples, and only a limited number focus on the grindability of densified products, which are the products most commonly comminuted in pulverized fuel power stations [28].

The aim of this study was therefore to assess a torrefied pellet using a series of experiments relevant to present and near future waste to energy conversion technologies. General characterization was accompanied by experimental tests using a modern small-scale downdraft gasifier system to give both a direct appraisal of performance in this type of energy conversion system, but also to provide evidence of feeding, material handling and durability characteristics which are relevant to other energy applications.

## 2. Material and methods

### 2.1. Materials

**Figure 1.**
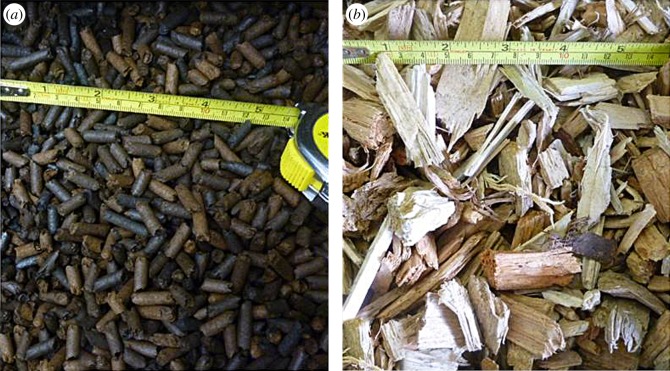
(*a*) Torrefied pellet and (*b*) P45 mixed wood chip used for gasification.

The torrefied pellets were produced using a proprietary process of microwave-induced pyrolysis in a continuous rotating bed reactor operating at around 175°C with a residence time of 15 min. Pellets were 6 mm diameter cylinders of average 5–15 mm length, formed from coniferous timber waste (saw dust; figure 1*a*). These were assessed in comparison to conventional untreated woody biomass chips, conventional torrefied pellet and untreated commercial biomass pellets. For gasification experiments, the wood chip was standardized to European specification P45 [25]. This comparator sample was produced from drum chipped mixed coniferous trees, acquired from the Midlands region of the UK (Nottinghamshire Ecofuels). Seasonal time of felling was unknown, other than within six months of supply and use (figure 1*b*).

### 2.2. Gasifier system

**Figure 2.**
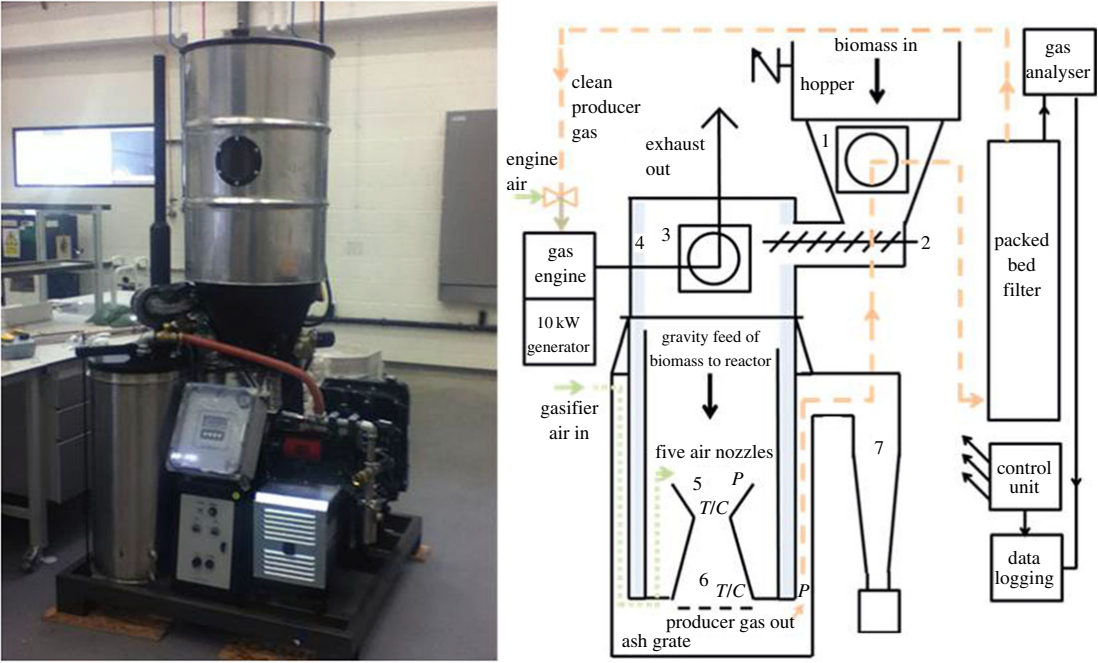
Ten kilowatt Power Pallet gasifier and schematic. (1) Drying bucket, (2) auger, (3) pyrocoil, (4) insulation, (5) combustion zone, (6) reduction zone, (7) cyclone particle separator. *T*/*C*, thermocouple and *P*, pressure sensors relay to control unit.

A 10 kW Power Pallet gasifier from GEK All Power Labs (USA) was used for experimentation. This comprised a conventional Imbert-style downdraft reactor (with 3^″^ throat diameter) supplemented with electronic controls and pre-heating process adaptations (figure 2). The system was designed for small-scale off-grid electricity production from wood chips, with feedstock size tolerances of 1.3 ≤ cm ≤ 3.8, and with less than 10% fines. The feedstock, once loaded into the hopper, was fed to the reactor by an automated 7 cm diameter smart auger, activated using an internal fuel-level paddle switch sensor. Within the auger channel (the drying bucket), there occurs non-contact heat exchange with post-reactor gases, and then secondly, non-contact heat exchange with engine exhaust in the ‘pyrocoil’ region situated directly above the gasification reactor. From the pyrocoil onwards, feedstock falls through the reactor under gravity. The system operates under slight negative pressure created during steady-state operation via a three cylinder Kubota spark ignition engine (although at start-up and shut-down, electric fan blowers are used to generate suction and to divert dirty gases from the engine to a flare stack via a manually operated valve). Sub-stoichiometric airflow enters the centre of the reactor and creates a small zone of combustion within (again via engine suction). The amount of air is sufficient only to provide heat for development of drying, pyrolysis and reduction zones and to create autothermal operation. For an explanation of downdraft gasifier thermochemistry and mechanics, see [6,16,51,52]. Thermocouples are placed at the base of the combustion zone and bottom of the reduction zone (upper and lower ‘T/C’ in figure 2), and there are two pressure sensors: one located at the combustion zone and the other after the reduction zone, to provide real-time readouts of temperature and pressure across the reactor. The resultant determination of pressure difference (*P*_ratio_) is linked to an intelligent grate shaker which activates when *P*_ratio_ falls below a set point. Gas is cleaned by a cyclone and a packed bed filter comprising replaceable wood chips, left over fines and a polishing stage of two oiled foam inserts.

The gas engine is connected to a generator rated at 10 kW_e_ and the system provides power on demand, meeting variations in external load by a governor controlled throttle. This consequently increases or decreases the rate of air (and gas) flow through the reactor. For these tests, a bespoke distribution box was provided (Distribution Zone, UK) and the electrical load was dumped into two 2 kW_e_ rated resisters (Cressall, UK).

### 2.3. Gasification product analysis

Producer gas analyses were extracted post filter (figure 2) and measured every 5 s using an online Gasboard 3100P by Wohun Cubic with inbuilt pump and adjustable flow meter set at 1 l min^−1^. Non-destructive analytical cells determined CO, CO_2_, CH_4_ and C_*n*_H_*m*_ by dual beam NDIR, and H_2_ by thermal conductivity. Raw gas was extracted post filter and passed through a water trap (filled with 350 ml of room temperature tap water), carbon filter and 0.3 µm polypropylene fibre F3000 (by CDK). The analyser was calibrated using high-purity bottled gases by STG.

Particulate and other condensable matter in the gas stream was captured in the water trap. Values were then quantified by gravimetric methods using a Buchner funnel and 70 mm QL100 filter paper by Fisher Scientific and an electronic mass balance (Ohaus Analytical Plus). This equates to the material entering the engine.

### 2.4. Gasification feedstock physical properties

Single species wood chip was also obtained from the same supplier (as §2.1), and represented the constituent parts of the mixed chip used in gasification experiments. The samples included both heartwood and bark. Three softwood species were assayed: European larch (*Larix decidua*), silver birch (*Betula pendula*) and Corsican pine (*Pinus nigra* ssp*. laricio*).

Bulk densities of the wood chip and torrefied pellet were measured using a volumetric cylinder and averaged over triplicate repeat sets. Moisture content was measured using a Dusiel digital moisture meter (probe) model MD812, from 20 samples. For all other analytical methods, the samples were reduced in size using a Retch ZM200 centrifugal rotor mill with a 0.50 mm cut stainless steel ring sieve. This produced a particle size for the biomass of 80% less than 0.25 mm.

#### 2.4.1. Proximate analysis

Proximate analysis was by thermogravimetry using a TA Instruments Q500, with samples placed into a clean platinum pan (10 mm diameter × 2 mm depth). Experimentation was at constant 1 bar pressure using two high-purity gases: 99.98% N_2_ and air, both from Air Products UK. Data were logged every 0.8 s and saved on a personal computer. From room temperature, the samples 24 ≤ mg ≤ 35 were heated to 110°C under N_2_ flow (of 120 ml min^−1^) at 10°C, and then held for 10 min. Temperature was increased to 900°C at a rate of 20°C ml min^−1^, and then held for 15 min. The carrier gas was then switched to air at a flow rate of 120 ml min^−1^.

#### 2.4.2. Ultimate analysis

A CE Instruments Flash EA1112 analyser was used to determine C, H, N and (by balance) O elemental compositions. Samples and standards were weighed (1.6 ≤ mg ≤ 3.8) for wood chip species, and (3.0 ≤ mg ≤ 3.3) for torrefied pellet, into tin capsules and then ignited inside the instrument at 900°C using oxygen over a copper oxide catalyst. Product gas was passed over electrolytic copper to remove O_2_ and magnesium perchlorate to remove H_2_O en route to a gas chromatography column and infrared detector. Helium was used as carrier gas. The analyses were repeated using a standard (2,5-bis(5-*tert*-butyl-2-benzo-oxazol-2-yl)thiophene) for elemental determination, weighed to a comparable value for each of the three types of sample.

#### 2.4.3. Calorific value

An IKA C5001 bomb calorimeter was used, set to dynamic method. Two tablets (combined weight 1.0092 g) of known calorific value were used. Benzoic acid (pelletized) IKA C723 was a pre-experimental standard. Pellets were made from the biomass samples by compressing the shredded materials, using a Greasby Specac manual press and a 13 mm stainless steel die. Compression was at 10 tonne pressure.

#### 2.4.4. Reaction kinetics

Using the TA Q500 instrument set up as previously (§2.4.1), a standardized methodology for downdraft gasification kinetics was used to determine parameters associated with reaction (2.1) [51]:

2.1 $$C+O_{2}\to CO_{2}\;\Delta H=-394\;\mathrm{kJ}\;\mathrm{mo}l^{-1}.$$

The reactivity of the sample under CO_2_ flow (2.1) was calculated using the common formula [53–57]:

2.2 $$k=\frac{1}{w_{0}}\frac{dw}{dt}.$$

From the derivative term of equation (2.2), *w* is the change in mass over time (*t*), in this case 12–24 s. *w*_0_ is *w*_t_ − *w*_daf_: the difference between the mass at the midpoint of the time interval, e.g. point at a tangent to the mass loss curve (*w*_t_) and end of reaction stage mass (*w*_daf_).

Gasification reactivity values were plotted against percentage conversion over the (2.1) stage. Kinetic values were then calculated based on conversions at 20%, between 1 and 30%, and between 5 and 50%. Extent of conversion was calculated from *X* = (*w*_i_ − *w*_t_)/(*w*_i_ − *w*_ash_), where *w*_i_ is the initial mass at the start of the reaction stage.

Kinetic parameters of (2.1) were determined by assuming that the reaction obeys the first-order Arrhenius equation, and by taking logs of both sides such that equation (2.3) takes the form of a linear equation on a Cartesian plot of ln(*k*) against 1/*T*, with *E*_a_/*R* as the slope and *A* as the *y*-axis intercept:

2.3 $$k=A\;\exp\left(-\frac{E_{a}}{RT}\right),$$

where *E*_a_ = activation energy, in J mol^−1^; *A* = frequency factor, in units of collisions s^−1^; *R* = universal gas constant, in J K^−1^ mol^−1^; *T* = temperature, in kelvin.

#### 2.4.5. Hygroscopicity

Simple water absorbance tests were completed on the torrefied pellet in comparison to commercially available standard pelletized miscanthus used for biomass boilers (from Terravesta, UK). Two glass measuring cylinders of 600 ml capacity were each filled with around 200 ml (116.51 ± 0.05 mg) of each pellet type. Simultaneously, 100 ml of room temperature tap water was poured into each of these and volume change was recorded at time intervals: 1 min, 2 min, 10 min, 1 h, 2 h, and 6 h. Moisture content of each pellet was measured before and after the tests.

#### 2.4.6. Grindability

The BWI for the torrefied pellets was determined using a dry grinding test in a standardized testing machine, the Bico Ball Mill [58]. The mill contained 285 steel balls of total weight 20.13 kg with a drum size of 305 mm in diameter by 305 mm in length which rotates at a constant speed of 70 r.p.m. The average diameter of 100 measured pellets was used as 80% passing feed particle size (*F*_80_) for the torrefied pellets in accordance with BS EN ISO 17829 [59]. The BWI test used 700 ml of dry sample (as per [60]) run for 100 revolutions in the mill, following which the contents were sieved to a set target equilibrium sieve size (*P*_1_). While the normal BWI test is defined on ascertaining the energy consumption in comminuting material to pass 100 µm, the target sizes used in full-scale coal mills for biomass and coal are different and based on the burner requirements. The target size was set to 1 mm based on pulverized fuel burner requirements for biomass [30,61]. The sieved fines were weighed and placed to one side, and new product was added to the oversized milled material to bring it back to its original weight. The new number of revolutions required was calculated from the results of the previous test to produce sieve undersize equal to 1/3.5 of the total charge of the mill. This process was repeated until the gram per revolution (*G*) reached a constant value for a minimum of three cycles. A full sieving analysis was performed on the last three cycles and the 80% passing size of the product (*P*_80_) was determined to calculate the BWI. The general form of the BWI equation is

2.4 $$W=10W_{i}\times \left(\frac{1}{\surd P_{80}}-\frac{1}{\surd F_{80}}\right),$$

where *W* is the work input and *W*_i_ is the BWI (both kWh t^−1^), which expresses the resistance of the material to crushing and grinding. *F*_80_ and *P*_80_ are the 80% passing size of the feed and product (µm), respectively. *W*_i_ can therefore be found through the following equation:

2.5 $$W_{i}=\frac{44.5}{{P_{1}}^{0.23}}\times G^{0.82}\times \left(\frac{10}{\sqrt{P_{80}}}-\frac{10}{\sqrt{F_{80}}}\right),$$

where *P*_1_ is the closing sieve size (µm) and *G* is the grindability (net g rev^−1^).

## 3. Results and discussion

### 3.1. Characterization

#### 3.1.1. Physical properties

The physical properties of the different feedstocks (table 1) reveal that the low-temperature microwave torrefied pellet had an increased fixed to volatile carbon ratio, and higher calorific value than the virgin wood from which it was derived. This phenomenon has previously been reported as being a consequence of the torrefaction process [23,24]. Bulk density was also 115% greater than the mixed wood chip. High calorific value and bulk density are beneficial properties for all fuel applications, as they equate to more energy per unit volume, and mean greater economy in fuel use, transportation and storage space. The relevance of fixed to volatile carbon ratio is discussed in §3.2.2.

**Table 1.** Proximate and ultimate analyses, gross calorific values and bulk density of samples (daf, dry ash free).

	ultimate analysis (% daf)				proximate analysis (% dry basis)				
sample	N	C	H	O	volatiles	fixed carbon	ash	calorific value (MJ kg^−1^)	bulk density g l^−1^
torrefied pellet	0.18	46.08	5.57	52.71	81.85	17.51	0.64	22.1	490
silver birch	0.11	49.28	6.05	53.37	85.5	13.9	0.6	18.4	227^a^
Corsican pine	0.19	48.91	6.31	47.17	83.6	15.7	0.7	19.0	
European larch	0.00	49.03	5.99	46.99	86.4	12.4	1.2	18.3	

a Mixed P45 wood chip.

#### 3.1.2. Reaction kinetics

**Figure 3.**
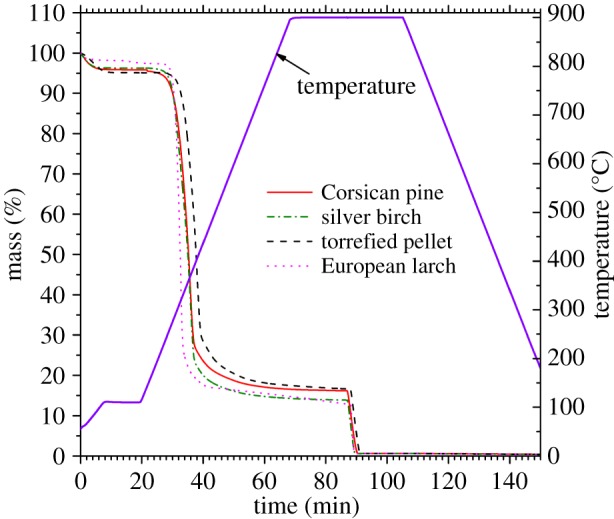
Results of thermogravimetric analysis for torrefied pellet and a range of virgin wood species.

The mass loss stages of biomass thermal decomposition have previously been well explored [20]. From figure 3, the second stage of thermal decomposition (onset approx. 22 min, up to completion at approx. 70 min) evidences pyrolysis. This reveals, and enables quantification of, the extent and rate of volatile release. The fixed to volatile carbon ratios seen here are in accordance with previous studies [51]. The mass loss at approximately 88 min occurs from the switch to 100% CO_2_ and therefore indicates the (2.1) reaction from which kinetic values were calculated. The depth of this mass loss stage also quantifies the amount of char (fixed carbon) by-product that would form within the gasifier. Although a high volatile content means a greater quantity of gas produced, to have a gas pure enough for engine supply requires a continuous region of char (formed from the feedstock) through which the pyrolysis gas passes en route out of the reactor. This char creates a ‘reduction zone’ and its purpose is to promote (2.1), and other reducing reactions [6,20]. No data exist on the optimum depth of this reduction zone, but although there are other influencing parameters, fixed-bed gasifiers are proved to operate well with feedstock fixed to volatile carbon ratios for those shown for the torrefied pellet in table 1 [6,19]. Differences between the species here would therefore influence subsequent reactor bed dynamics by the extent of carbon available which would ultimately affect the quality of gas produced.

**Figure 4.**
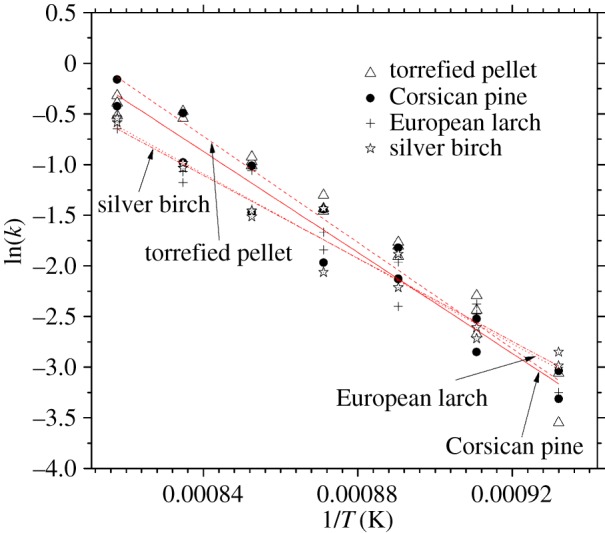
Kinetic parameter ln(*k*) as a function of 1/*T* for all samples after isothermal experiments. Plots shown were taken over mass loss stage 20% conversion. All plots 0.95 ≤ *R*^2 ^≤ 0.96.

Although fixed-bed gasifiers are autothermal and therefore do not require external heat input to maintain equilibrium, knowledge of relative activation energies is still practically important for reactor sizing and to optimize fuel conversion efficiency. The torrefied pellet exhibited a linear trend on a Cartesian Arrhenius plot congruent to those of other wood species (figure 4). This is not surprising considering that its molecular composition was likely one or all of the same species, in particular pine, which predominates as a softwood building material and which most closely compares to the torrefied pellet in this study's results (see also table 2). These results suggest that the torrefaction process had not significantly altered inherent kinetic properties to a great extent, despite both activation energy and pre-exponential factor being just slightly higher than virgin pine.

**Table 2.** Activation energies and pre-exponential factors at varying conversion levels.

	*X* = 20%		*X* = 1–30%		*X* = 5–50%	
sample	*A* (min^−1^)	*E*_a_ (kJ mol^−1^)	*A* (min^−1^)	*E*_a_ (kJ mol^−1^)	*A* (min^−1^)	*E*_a_ (kJ mol^−1^)
torrefied pellet	1.74 × 10^9^	217.8	4.96 × 10^8^	205.4	1.59 × 10^9^	218.1
silver birch	9.41 × 10^6^	170.1	1.13 × 10^6^	159.0	1.29 × 10^7^	174.2
Corsican pine	5.19 × 10^8^	207.2	1.85 × 10^8^	197.0	1.14 × 10^9^	216.0
European larch	2.98 × 10^7^	180.6	1.05 × 10^7^	170.2	1.58 × 10^7^	176.1

**Figure 5.**
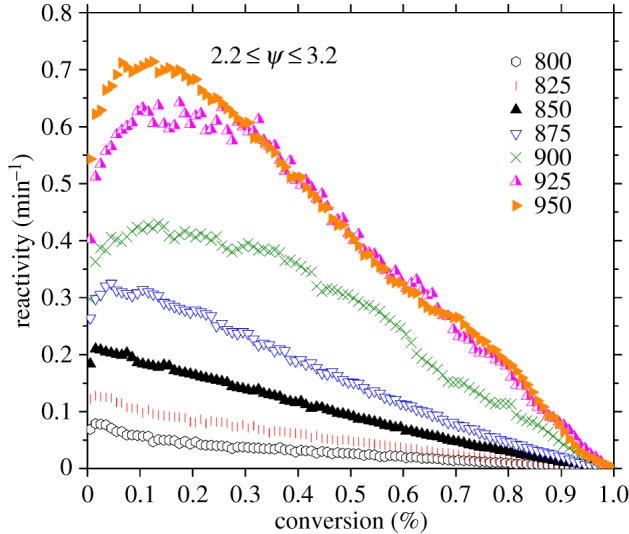
Reactivity as a function of conversion for Rotawave torrefied wood pellet. Data points shown selected at 0.01% conversion interval. Peak fitting at *R*^2^ = 0.99 was used to identify *χ*_max_. *Ψ* correct to ±0.1.

Plots of reactivity as a function of conversion for the torrefied pellet match well with the theoretical predictions of the random pore model [62], which represents particle thermolysis as developing by a random overlapping of cylindrical voids that grow as a function of temperature from the initial pores. The random pore model introduces a structural parameter term (*Ψ*) which defines the initial physical properties of the particle, and which determines the shape of the reactivity versus conversion curve. Increasing value for the structural parameter is associated with reactivity versus conversion curve convexity, which derives from progressive particle porosity [63]. These values (shown in figure 5) were calculated from the peak of conversion as a function of reactivity using equation (3.1) [64]:

3.1 $$\Psi =\frac{2}{\left[2\;\ln(1-\chi_{\max})+1\right]}.$$

Sixth-order polynomial curves were fitted. These torrefied pellet curve shapes and values for *Ψ* closely match those previously reported for all European softwoods, and again in particular Corsican pine [51]. This again suggests that the torrefaction process had not changed the biomass internal structure with regard to porosity development. The relevance of which is that porosity determines the availability of surface sites for chemical reactions, and therefore a gasifier should be able to tolerate switching between chipped wood and torrefied pellet in this respect.

#### 3.1.3. Hygrosopicity

When compared against standard miscanthus pellet (initial moisture 1.6%), the miscanthus pellet had swollen by 50% after 1 min, 100% after 2 min and 150% after 1 h, whereas the torrefied pellet (initial moisture 0.2%) remained unchanged. After 24 h, the torrefied pellet was removed from the water, and it had not changed in volume or weight, whereas the miscanthus pellet had completely lost all its shape and form. This is a highly advantageous generic property of the torrefied pellet in terms of handling and storage for all fuel applications as it reduces the need for measures to protect against moisture ingress. For all bio-energy applications, moisture absorbance greatly reduces the calorific value of a fuel, because of the high parasitic enthalpy demands of H_2_O inside the reactor. For small-scale downdraft gasification, this property has extra significance, as it would overcome the need for a full clean out of the reactor bed after each shut-down due to pellet disintegration.

### 3.2. Gasification tests

#### 3.2.1. Wood chip

Downdraft gasification technology has a rich history, and it is proven to work on chipped or chopped wood feedstock, although it is a fact that there are many instances of system failures [6]. It was not therefore the aim to test whether or not the system would work with wood chip, rather that the results should provide a comparative appraisal of qualitative and quantitative performance. Notwithstanding this, no peer reviewed literature could be found that reported the efficacy of this type of modern gasifier.

Long duration stable operation of the gasifier was achieved but only by screening the wood chip (7 mm cut) of fines. Excessive pressure drop across the reactor otherwise occurred. Sieving of the wood chip was also necessary to remove excessively large or elongate pieces which were found to cause both bridging and auger jams. This was laborious work, and highlights the need for feedstock uniformity. It also evidences the lack of market structure at present with respect to the availability of suitable feedstock for gasifiers, as the P45 grade was the closest match to the system design specification. Sustainable woody biomass waste that is produced from arboriculture at the local scale in Europe is presently chipped for size reduction and ease of removal rather than for use as an energy commodity, and in particular one which is suitable for gasification (low volume of fines and narrow range of chip sizes). But, this is not an insurmountable challenge, and 80 years ago when gasification use was at its peak, some countries actually had a well-developed national infrastructure for feedstock supply through garages and filling stations [6]. The absence of such an infrastructure at present would however hold back current system penetration.

**Figure 6.**
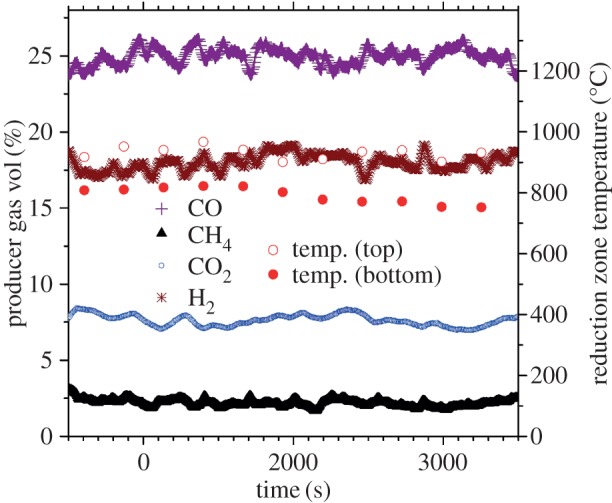
Producer gas output from P45 mixed wood chip, and reduction zone temperatures. System running at steady state providing 4 kW_e_, representative of all experiments. C_*n*_H_*m*_ values (not shown) were 2 ≤ ppm ≤ 5. Temperature standard deviation about the 2 h mean = 19°C (top) and 22°C (bottom). Gas analyses accuracy less than or equal to ±2% full scale, less than or equal to 2 repeatability.

At switch on, the system took approximately 30 min to reach satisfactory temperature for engine ignition (around 850°C for the top thermocouple and 700°C for the thermocouple at the base of the reduction zone). The system ran well at output levels of 4 kW_e_, and 2 kW_e_. Once the engine was operational, temperature remained stable (other than small oscillations) and in the expected range for all runs, and consequently, gas composition of H_2_ and CO was relatively stable also (figure 6).

The particulate matter collected from the gas analyser water trap for all runs was of 48 ± 9.8 mg Nm^−3^. For long-term producer gas supply to an internal combustion engine, the maximum concentration of tar and particulates is suggested as being 50 mg Nm^−3^ [6,65]. Online gaseous hydrocarbons (C_*n*_H_*m*_) were detected in the range of 0 ≤ ppm ≤ 10 at steady-state experimentation, and so considered negligible. Water will be expected to capture all the particulates, gravimetric tars, polar tars, and up to 39% of non-polar tars [66], but the values obtained here must still be considered as a minimum. The higher than recommended fraction of small pieces (fines included) contained within the feedstock can perhaps explain why the particulate and tar concentration was around the maximum suggested value, due to how small pieces inhibit free gas transfer within the reactor [20].

One benefit of the wood chip filter was that it also cooled the gas prior to its entry into the engine manifold. Colder gas means greater energy density per unit volume. Approximately after 100 h of operation, the filter was checked and apart from the bottom few centimetres, it appeared free of tar. These cheap passive filters have the advantage over wet scrubbers which create a hydrocarbon-rich wastewater and therefore potentially high disposal costs for the lifetime of the system.

#### 3.2.2. Torrefied pellet

Preliminary experiments were run to populate the reactor combustion and reduction zones with torrefied pellet char. To this end, the reactor chamber was loaded with lumpwood charcoal in size range of 1.5 ≤ cm ≤ 5 (from Big Green Egg, UK) to just above the air inlet (top of combustion zone). The hopper was removed and the torrefied pellet was observed as it was fed through the auger system into the pyrocoil. The angle of the hopper feeding channel was 30° from vertical, and the pellet was seen to have excellent flow characteristics (electronic supplementary material, S1), due to its cylindrical shape and smooth, hard surface texture. This is beneficial for all automated feeding systems. For small-scale gasification, it has the added bonus of alleviating common issues of bridging and channelling.

The gasifier reactor was then run for 2 h at steady 2 kW_e_ load, to move the torrefied pellet through to the combustion and reduction zones. Close to the end of this preliminary test, there was a build up of internal pressure notifiable by repeated activation of the *P*_ratio_ alarm sensor. This was manually quietened since the duration of the preliminary test was almost complete.

**Figure 7.**
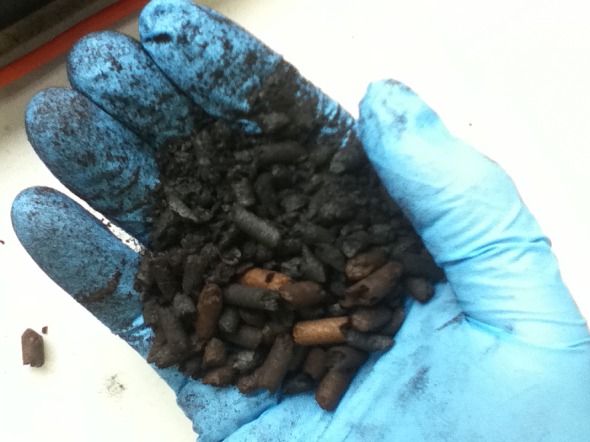
Torrefied pellet extracted from the combustion zone of Power Pallet gasifier after failed experimental run.

The first full test started well, with instant ignition and a very clear blue flame from the flare stack evidencing a gas free of tar and soot. Before the engine could be brought in-line, reactor temperature quickly reached above safe operating limits (*T* > 1050°C) and was still climbing. Afterwards, upon examination of the cooled reactor, a high concentration of pellet dust was found inside—the likely cause of the high pressure while running the preliminary test (figure 7). High temperature can be beneficial inside a downdraft gasifier as the desired reactions are promoted. The rate of (2.1) in particular drops rapidly below 800°C [51]. Yet, too high a temperature can lead to spreading combustion which therefore decreases the size of the reduction zone. In addition, very high temperatures can cause internal damage to the air nozzles [7,20], and also lead to the formation of fused deposits inside the reactor [67]. The reactor was cleaned out and re-filled with the torrefied pellet char (minus the fines).

Numerous repeated attempts were made with the same result: rapid increases in temperature, along with increased reactor pressure drop. Sometimes the combustion zone would ignite, but the flame in the flare stack would not, being very smoky and extinguishing itself regardless of gas and air mixing levels. These occurrences indicated that packing in the pyrocoil was loose, but that the reduction zone/throat was densely packed with pellet dust. This proved to be correct each time the reactor was cleaned out.

With the reactor switched off, the hopper removed and the pyrocoil viewport open, observations were made on the feeding properties of the torrefied pellet from the reactor side of the auger channel. It was clearly being partially crushed in the base of the channel as the auger rotated. This grinding action generated a quantity of fine powder which fell down into the reactor as the pellet was being fed through. The powder then created high-pressure build-up in the reactor bed, leading to inadequate gas and heat transfer, and consequently poor gas quality. Although frequency of grate shaking was increased, this did not rectify the problem, and the only option was to fully empty the reactor. In general handling of the pellet, and while monitoring its flow above the auger channel, no dust was observed to form.

With regard to the high reactor temperatures, this was undoubtedly due to the high calorific value and energy density of the torrefied pellet. Both highly attractive properties, but for a downdraft gasifier set up to operate within a relatively rigid range of feedstock moisture, the torrefied pellet could not be accommodated. This gasifier system did not have in-built adjustments to permit the required changes, and indeed it highlights the limitations that presently exist with this technology. Even a modern system must still be considered a rigid technology where once set up to operate at a certain feedstock parameter, there is limited flexibility to tolerate feedstock variations which then result in excessive maintenance requirements, and lower than specified performance [16,24,26]. Of note however is that other downdraft gasifiers are gravity rather than auger fed, with the feedstock hopper situated directly above the reactor [16]. Chemically, the torrefied pellet would appear to be a highly attractive gasifier feedstock, and one which could be able to make use of a variety of otherwise unsuitable waste biomass materials. This, along with the likely lower NO_*x*_/dioxin emissions, overall efficiency and financial gains, and greater CO_2_ mitigation potential, makes the combined technologies in many ways far more attractive for future commercial development than traditional grate combustion. The small size of the pellets may be problematic long term as even without being subjected to attrition in the auger channel, the size was at the low end of design tolerance for these systems. It would not seem overtly challenging to produce a larger sized, torrefied pellet, and a useful future study would then be to re-attempt these tests in a standard gravity fed downdraft gasifier. Notwithstanding the above, the torrefied pellet friability under attritional stress is a beneficial attribute for many combustion applications.

### 3.3. Grindability

Owing to the hydrophobic nature of the torrefied pellets, it was not possible to obtain the common particle size distribution of disintegrated pellets [68]. Thus, it was not possible to use the particle size comparison as a matrix of biomass grindability in comparison to several previous studies [40–44]. The HGI test uses small masses or volumes [33,34,41,69], and thus for biomass pellets, the BWI test offers a grindability test with a more representative volume (700 ml compared to 50 ml), allows for variable target sizes, and for the pellet diameter to be used as the initial feed size.

The BWI (*W*_i_) expresses the resistance of the material to grinding to a specified product size, and the higher the value of *W*_i_, the more difficult the material is to grind to the required product size. The work input *W* gives the grinding power required by the mill as described. These results are illustrated in table 3 accompanied by the results of a previous study on the application of the BWI test on a range of densified biomass pellet [38]. The low-temperature microwave torrefied pellet performed favourably in the BWI test compared with untreated and other thermally treated biomass with a *W*_i_ of 25 kWh t^−1^. Several non-treated biomass pellets exhibited mill choking (mixed wood, miscanthus and sunflower pellets), which resulted in very high *W*_i_ values (413, 426 and 366 kWh t^−1^, respectively), but this was not observed for the torrefied pellet. The torrefied pellet performed on a par with another commercially produced torrefied pellet (denoted as traditional torrefied pellet) which had a *W*_i_ of 16 kWh t^−1^. This traditional torrefied pellet was produced through the conventional torrefaction method of heating virgin wood chips in an oxygen-free environment. Interestingly, both torrefied pellets showed significantly lower BWI values than the steam exploded pellets, which had a much higher *W*_i_ of 64 kWh t^−1^. This is due to the product particle size of the steam exploded pellet being very small (*P*_80_ of 355 µm), while the torrefied pellets had *P*_80_ sizes much closer to non-treated biomasses (758 µm for the traditional torrefied pellet and 881 µm for the microwaved torrefied pellet). As with the traditional torrefied pellet, the milling energy required to reduce the pellets to the target particle size of sub 1 mm was a low percentage of the calorific value of the microwave torrefied pellet at 0.08%.

**Table 3.** BWI results for microwave torrefied pellets. *F*_80_, 80% passing feed size; *P*_80_, 80% passing product size; *G*, grindability per revolution; *R*_F_, final revolution count; *W*_i_, BWI; *W*, work input; *H*, gross calorific value on dry basis; *W*/*H*, work input-gross calorific value ratio.

material source	*F*_80_ (µm)	*P*_80_ (µm)	*G* (g rev^−1^)	*R*_F_	*W*_i_ (kWh t^−1^)	*W* (kWh t^−1^)	*H* (kJ g^−1^)	*W/H* (%)
this study								
torrefied pellets	5970	881	2.015	62	25	5	22.1	0.08
source [32]								
wood pellets	8400	786	0.053	2141	413	102	20.4	1.80
miscanthus pellets	6290	811	0.057	2168	426	96	18.6	1.86
sunflower pellets	8620	764	0.059	1699	366	93	20.2	1.66
eucalyptus pellets	8390	757	0.340	411	87	22	19.8	0.40
steam exploded pellets	5910	355	0.283	556	64	26	20.0	0.46
traditional torrefied pellets	8000	758	2.655	60	16	4	21.8	0.07
olive cake	3712	590	0.202	390	136	34	19.3	0.63
La Loma coal	2709	77	0.664	242	23	22	30.0	0.26

## 4. Conclusion

This study assessed the performance of a torrefied wood pellet produced by low temperature microwave pyrolysis through a series of experiments to assess its suitability as an energy feedstock. Tests with a modern small-scale downdraft gasifier in comparison to wood chip at standard specification were combined with characterization relevant to other industrial fuel applications.

The torrefied pellet was found to have the following beneficial attributes as an energy feedstock:

1. Relatively high calorific value and high bulk density in comparison to virgin wood chip.
2. High water repellence, which would allow it to be stored outdoors or indoors in high moisture areas. For downdraft gasification, this would overcome issues of steamed disintegration.
3. Significant improvement in grindability compared to other non-treated and thermally treated biomass pellets. The low grinding energy and comparable particle size with non-treated biomass pellets in the BWI test suggest good particle size control which would make it a suitable fuel for pulverized fuel power stations, or fluidized bed gasification systems.
4. The torrefaction process did not appear to have altered inherent kinetic properties; however, both activation energy and pre-exponential factor (for CO_2_ gasification) were slightly higher than original virgin biomass.
5. Flow and gravity feeding properties were found to be excellent.

The torrefied pellet proved to be practically unsuitable as a feedstock in this modern gasifier:

6. Its high calorific value and energy density resulted in an excessively high reactor temperature.
7. The auger feeder was the main detrimental component, as it partially crushed the pellet to a fine powder. This in turn blocked the reactor core. In general handling of the pellet and while observing its flow inside the hopper, no dust was observed to form.
8. For the above reasons, steady-state operation could not be achieved with the 10 kW_e_ Power Pallet. Yet, the torrefied pellet was found to have properties that could alleviate some of the challenges presently restricting the increased penetration of small-scale downdraft gasifiers (points 1, 2, 4 and 5). Consequently, there is a strong case for continuing research in torrefied pellet as a gasifier fuel, and for research with a gravity fed downdraft gasifier. The problems encountered were those commonly associated with downdraft gasifiers which are not set up for this type of feedstock, and which do not permit the necessary means of simple operator adjustment.

## Data accessibility

Data available from the Dryad Digital Repository: http://dx.doi.org/10.5061/dryad.nn8pj.
